# Effectiveness of Single-Use Negative Pressure Wound Therapy (Pico 7) in Chronic Lower Limb Wounds: A Randomized Clinical Trial

**DOI:** 10.3390/jcm15166373

**Published:** 2026-08-18

**Authors:** Celia Villalba-Aguilar, José Alberto Laredo-Aguilera, Lucía Villalba-Aguilar, Esperanza Barroso-Corroto, Cristina Del Viso-Cudero, Víctor Serrano-Fernández, Juan Manuel Carmona-Torres

**Affiliations:** 1 Hospital Laboral Solimat, 45004 Toledo, Spain; celia.villalba@alu.uclm.es; 2 Facultad de Fisioterapia y Enfermería de Toledo, Universidad de Castilla-La Mancha, 45071 Toledo, Spain; esperanza.barroso@uclm.es (E.B.-C.); juanmanuel.carmona@uclm.es (J.M.C.-T.); 3 Grupo de Investigación Multidisciplinar en Cuidados (IMCU), Universidad de Castilla-La Mancha, 45071 Toledo, Spain; 4 Instituto de Investigación de Castilla-La Mancha (IDISCAM), 45004 Toledo, Spain; 5 Facultad de Ciencias de La Salud, Universidad Rey Juan Carlos, 28922 Alcorcón, Spain; luciavillalbaaguilar12@gmail.com; 6 Hospital Universitario de Toledo, 45007 Toledo, Spain; delvisocudero@yahoo.es; 7 Departamento de Enfermería, Facultad de Medicina, Universidad Autónoma de Madrid, 28029 Madrid, Spain; victor.serrano@uam.es

**Keywords:** negative pressure wound therapy, diabetic foot ulcer, vascular ulcer, wound healing, nursing, PICO 7

## Abstract

**Background:** Chronic lower limb wounds, such as diabetic and vascular ulcers, affect up to 2% of the global population, imposing high healthcare costs and a significant risk of amputation. Although nursing care and traditional treatments are fundamental, single-use portable Negative Pressure Wound Therapy PICO 7 system, has emerged as a cost-effective alternative. The objective of this study was to evaluate the effectiveness of PICO 7, compared to the usual treatment in chronic lower limb wounds or their dehiscence in amputations. **Methodology:** A randomized, controlled, open-label clinical trial was conducted at the Hospital Universitario de Toledo (2024–2026). The final analyzed sample consisted of 31 patients (Intervention Group, IG: 14; Control Group, CG: 17). Both groups followed the dynamic TIME strategy to standardize wound bed preparation. Primary variables included healing rate, healing time, wound area reduction, clinical safety (infections and adverse effects), pain levels, health-related quality of life, and physical activity. Statistical analysis was performed using SPSS v.28. **Results:** The IG showed a higher mean percentage reduction in wound size, although the between-group difference did not reach statistical significance. No infections were recorded in the IG. Regarding quality of life and physical activity, the CG showed a significant post-intervention decline, whereas the IG remained stable. A significant negative correlation was found between final wound size and hours of sleep, suggesting that wound reduction facilitates better rest. **Conclusions:** The PICO 7 system appears to be a safe and feasible alternative to conventional wound care. Favorable trends in wound area reduction should be interpreted cautiously given the lack of statistically significant between-group differences.

## 1. Introduction

Chronic wounds of the lower limbs (LLs) have an incidence of 1–2% of the world’s population, making them the most common chronic wounds [1,2]. These wounds include diabetic foot ulcers (DFUs) and vascular foot ulcers (VFUs), both arterial and venous, which are associated with high morbidity, risk of amputation, and high healthcare costs [1,3,4].

Currently, DFUs represent one of the most prevalent and serious complications; according to the report from the International Diabetes Federation (2025), the global prevalence has already reached 589 million adults [5,6,7]. These lesions are associated with a 2.5 times higher mortality rate and are responsible for up to 85% of non-traumatic amputations in aging populations such as Spain’s [8]. According to studies conducted in Europe, a patient with a DFU incurs a cost around €5000, accounting for up to 3% of total healthcare expenditure [9,10].

Regarding VFU, at least 70% are the result of chronic venous insufficiency (CVI), appearing in 5% of the elderly population [1,8,11]. Chronic venous disease (CVD) represents a critical financial burden for healthcare systems in Western Europe, with an annual expenditure of between 2% and 4% of the total health budget. VFUs are the ulcers with the highest annual expenditure, with an average cost of €9000 per patient per year [8,12,13].

Several factors, such as Diabetes Mellitus (DM), peripheral arterial disease (PAD), smoking, and obesity (reflected in an increase in body mass index (BMI)), have been associated with impaired wound healing and its tendency to chronicity [14,15,16,17]. In this context, the need for surgical interventions and amputations in patients with DFU and VFU carries a high risk of surgical wound dehiscence, a frequent complication that increases morbidity and delays definitive recovery [6,16,18,19]. Additionally, the anatomical location of the lesion, especially in areas of low perfusion or subjected to pressure, and the patient’s pharmacology, which may interfere with platelet function and the inflammatory response, play a role [16]. Consequently, these wounds often require long-term treatments and repeated healthcare interventions, prolonging hospital stays and significantly increasing healthcare system costs [16,20,21].

The care of these wounds is one of the competencies of nursing professionals [22], and it primarily focuses on optimizing the patient’s well-being by avoiding or alleviating external forces that worsen or hinder healing, such as pressure, shear, or moisture, while considering nutritional support, wound care, and infection prevention [22,23]. In the conventional treatment of ulcers, different alternatives are used, such as traditional dressings, surgical or enzymatic debridement, oxygen therapy, hydrotherapy, compressive therapies, microcurrent-based therapies, or platelet-rich plasma (PRP) [24]. However, the Spanish Society of Vascular Surgery highlights the combination of these methods with negative pressure therapy (NPWT) as an effective complementary option [1]. This technique, approved by the Food and Drug Administration (FDA) in 1997 [11], involves an adjuvant mechanical treatment that accelerates healing by draining excess exudate and edema, eliminating barriers to cell proliferation, and can be used continuously or intermittently [25,26]. Its implementation promotes the formation of granulation tissue and reduces bacterial load [1]. Although its efficacy is notable in chronic wounds, its use has successfully expanded to acute injuries, burns, or surgical dehiscence, where evidence supports its ability to optimize healing and reduce infection risk [27,28].

Currently, single-use portable NPWT systems are available to facilitate wound care in outpatient and home settings. Among them, the Avelle™ system (Convatec Inc., Reading, Bershire, UK) and the PICO™ system (Smith & Nephew plc, Hull, UK) (with continuous pressure of −80 mmHg) stand out, which use their absorbent dressing technology without a rigid reservoir and operate on batteries; or the manual activation system SNAP™ (3M Health Care, St. Paul, MN, USA) (−125 mmHg). These models optimize patient autonomy by being portable, ultra-lightweight, and silent devices, specifically designed for small, low-exudate wounds that require less frequent dressing changes, thus promoting treatment adherence outside the hospital environment [1,29,30].

The single-use portable NPWT system (PICO™) has proven to be highly effective in treating chronic, hard-to-heal lower limb wounds [31,32]. This system not only accelerates healing and improves the patient experience but also optimizes healthcare management by reducing readmissions, dressings, and costs [5], with savings estimated by The National Institute for Health and Care Excellence (NICE) of up to £101 per patient in trauma surgeries [15,16]. In the Spanish context, the International Diabetes Federation endorses its indication due to the alarming increase in the number of amputations in recent years, consolidating this technology as a cost-effective investment that offsets its cost through faster recovery [7].

Although single-use portable NPWT systems (such as PICO™) have shown promising clinical utility, existing evidence remains substantially limited. Current randomized controlled trials (RCTs) evaluating single-use NPWT in chronic lower extremity wounds suffer from notable methodological constraints, including small sample sizes, brief follow-up periods, heterogenous populations, and a predominant focus on closed surgical incisions or acute trauma rather than complex chronic ulcers. Furthermore, high-quality RCT evidence specifically addressing single-use NPWT in southern European populations, where healthcare delivery models and outpatient nursing workflows differ markedly, is scarce [1,29,33].

A fundamental distinction must be drawn between traditional NPWT systems and single-use portable NPWT devices such as PICO 7 [1,29,30]. Traditional NPWT relies on heavy, electromechanical pump units with rigid canister reservoirs requiring frequent clinical monitoring, continuous power supplies, and bulky tubing. These features often restrict patient mobility, increase hospital length of stay, elevate healthcare costs, and cause significant physical and psychological disruption to daily living [29,30]. In contrast, single-use portable systems utilize canister-free, ultra-lightweight, battery-operated technology with high-absorbency dressing materials that manage exudate via evaporation and absorption [1,29]. This design offers key advantages: enhanced patient mobility, discretion, continuous outpatient compliance, reduced nursing workload, and cost reduction by minimizing readmissions and dressing changes [22,31,32]. However, single-use systems also present specific technical limitations, such as a lower fixed negative pressure (−80 mmHg compared to −125 mmHg for traditional systems) [1,34], potential saturation issues in highly exudative wounds, and reliance on proper airtight seal maintenance by outpatients in home-based settings [29,34].

Given that the efficacy of NPWT mechanisms in wound bed preparation is already established in broad clinical literature [27,34], the present trial was not designed to demonstrate absolute clinical superiority over conventional therapy. Instead, this study primarily aimed to evaluate the pragmatic feasibility, clinical safety, and real-world performance of the single-use portable NPWT system (PICO 7) within a specialized outpatient vascular nursing setting.

We hypothesized that implementing single-use portable NPWT (PICO 7) in real-world clinical practice represents a feasible, safe, and effective therapeutic approach for chronic lower limb wounds and post-amputation dehiscence, yielding comparable or favorable healing trajectories, low complication rates, and preserved quality of life relative to standard care.

The primary objective was to evaluate the feasibility and clinical safety of PICO 7 compared to conventional advanced wound care in patients with vascular foot ulcers, diabetic foot ulcers, or surgical dehiscence. As a specific objective, it aimed to analyze the therapeutic outcomes in terms of healing rate and time, patient safety, pain management, and quality of life, considering the influence of comorbidities such as DM, obesity, and smoking.

## 2. Methodology

This section describes the methodological framework employed to evaluate the clinical effectiveness and safety of single-use portable NPWT (PICO 7) compared to standard care. Specifically, it details the study design and clinical setting, participant eligibility and recruitment criteria, sample size determination, intervention protocols, outcome measures, and statistical analysis procedures applied.

### 2.1. Design

A randomized, controlled, open-label clinical trial was conducted in a nursing consultation of the Vascular Surgery Laboratory at the Hospital Universitario de Toledo from January 2024 to January 2026. An open design was chosen due to the physical nature of the NPWT (PICO 7) medical device used, which prevents masking of the intervention. This study was registered on ClinicalTrials.gov with registration number NCT05877378 on 26 May 2023.

The participants were divided into two study groups: the experimental group (IG) treated with PICO 7 NPWT, and the control group (CG), treated conventionally.

In both groups, the dynamic TIME strategy was followed to optimize the wound bed and reduce factors that delay healing. This protocol was based on the control of non-viable tissue (T), the management of inflammation and infection (I), moisture balance through exudate control (M), and stimulation of epithelial edges (E). The objective of applying this common approach was to standardize baseline care, ensuring that the reduction of edema and bacterial load was consistent across all participants [27].

### 2.2. Subjects and Study Scope

The reference population consisted of male and female patients over 18 years of age residing in the province of Toledo, who attended the vascular surgery laboratory of the Hospital Universitario de Toledo and presented with a DFU, VFU, or dehiscence of surgical wounds that met the inclusion criteria.

### 2.3. Inclusion and Exclusion Criteria

The inclusion criteria included subjects of both sexes over 18 years of age presenting with a vascular pathology (arterial or venous) with a DFU or VFU, as well as patients with surgical dehiscence in the lower limbs. Wounds with a size smaller than 20 cm^2^ and patients with associated risk factors such as smoking, DM, obesity, or HBP were included. Additionally, the ability to comply with the protocol and the signing of informed consent were mandatory requirements.

On the contrary, exclusion criteria were established as the presence of malignant ulcers or with abundant exudate, and anatomical locations that prevented an airtight seal of the dressing. Patients with known allergies to NPWT components, pregnancy, severe cardiovascular diseases, vasculitis, claudication, or active diagnoses of Charcot foot were also excluded. Systemically, subjects undergoing chemotherapy or corticosteroid treatment, those who received NPWT or hyperbaric oxygen in the previous seven days, or who presented hematological alterations (leukopenia, thrombocytopenia, anemia, or liver dysfunction) were excluded. Finally, patients with deep vein thrombosis, malnutrition, eating disorders, or exposure of deep structures such as muscles, tendons, and bones were not eligible.

### 2.4. Sample Size

Taking as reference the article by Kirsner R et al. in which the single-use NPWT system was compared with traditional NPWT, there was an average percentage reduction in wound size at 12 weeks of treatment of 90.24% for the PICO 7 system (Smith & Nephew Medical Ltd., Hull, UK) and 51% for conventional NPWT [1]. For the sample calculation using the Grammo version 12 program (Institut Municipal d’Investigació Mèdica, Barcelona, Spain), an alpha risk of 0.05 and a beta risk of 0.2 in a bilateral contrast were accepted, requiring 15 subjects in the first group (IG) and 15 in the second group (CG) to detect a statistically significant difference between two proportions, which for IG was expected to be 0.9 and for CG 0.5. The ARCOSINE approximation was used. However, 20 subjects were included in each group to anticipate possible losses.

The required sample size assumed a two-tailed alpha level of α=0.05 and a statistical power of 80% (1−β=0.80), accounting for an anticipated 15–20% drop-out rate. However, participant attrition due to loss to follow-up and mortality resulted in a final analytical n=31 (n=14 in the IG and n=17 in the CG), which limited the post-hoc statistical power to detect minor between-group differences.

### 2.5. Study Variables

The outcome variables of this study were first measured before the start of the trial (T0) and then each time the dressing was performed (T1, T2…T12).

For the development of this study, the following independent variables were included:

- Sociodemographic variables: sex, age, employment status, and nutritional status.
- Physical activity through the International Physical Activity Questionnaire (IPAQ) [35], taking usual treatment.
- Anthropometric variables: weight, height, body mass index, and hours of sleep.
- Preexisting medical conditions: number of lesions, DM, smoking habit, degree of obesity, hypertension (HBP), dyslipidemia (DL), peripheral vascular disease (PVD), congestive heart failure (CHF), rheumatoid arthritis, osteoarthritis, deep vein thrombosis (DVT), varicose veins, anemia, immunodeficiencies, and neuropathies.
- System PICO 7: hours of use, anatomical location, and functional failures.
- Characteristics of Chronic Wounds: Location and Etiology (VFU or DFU). For the latter, the classifications of Rutherford [36] and Wagner [37] were used, respectively, in addition to recording surgical wound dehiscence due to amputations caused by these wounds.

The following dependent variables were also analyzed:

- Wound healing trajectory: wound size measured using a paper ruler and the Healico application (from Urgo Medical), healing time, and total number of wounds with complete healing.
- Clinical safety: adverse effects of both treatments (occurrence of perilesional pressure ulcers, increase in wound area, necrosis, maceration or bleeding, hypersensitivity to materials, infection confirmed by exudate culture or strong odor, leaks), changes in pain measured by the Visual Analogue Scale (VAS) [38], and use of analgesics.
- Health-related quality of life, assessed using the EuroQol-5D or EQ-5D scale [39].

### 2.6. Data Collection Instruments

Regarding the data collection instruments, the following were used:

- Medical records of the patients participating in the study.
- Healico© (Urgo Medical, París, Francia) application photo analysis [40]: used to measure the size of the wound. This application consists of software for analyzing wound images and calculating their area.
- Data collection form for patients, which includes an initial assessment and the questionnaires mentioned below. This form collected general patient data, habits and the physical activity they engage in, as well as diseases of interest for this study and health-related quality of life (HRQOL):
  o  International Physical Activity Questionnaire (IPAQ). This questionnaire consists of 7 questions about the frequency, duration, and intensity (moderate and vigorous) of physical activity performed in the last seven days, as well as walking and time spent sitting on a workday [35]. It is worth noting that performing physical exercise causes the contraction of the calf muscles, promotes the compression of the veins, and increases venous pressure, promoting venous return. Therefore, people who engage in physical exercise will have positive effects on the healing of VFU and DFU [41,42].
  o  The health-related quality of life questionnaire for patients with chronic wounds used was the EuroQol-5D or EQ-5D scale. It consists of two parts. The first part is a questionnaire where the state that best describes current health is marked. The second is the VAS pain scale, which reflects overall health status in terms of pain [39].
- Form for completion by nursing staff, where they recorded the clinical progression of the injury.

### 2.7. Procedure

Patients were recruited in the vascular surgery laboratory of the Hospital Universitario de Toledo. Detailed information about the study was provided to all patients who attended the consultation with at least one chronic wound in the lower limbs (including dehiscence, pressure ulcers, and DFUs) and who were diabetic, smokers, and/or obese.

Assignment to the groups (IG and CG) was performed randomly using a random number table generated by researcher JMCT. Following the order of arrival, the information sheet and informed consent were given to patients who met the inclusion criteria. Those who agreed to participate completed a self-developed form and the EuroQol-5D scale [39].

For both groups, the predefined primary endpoint of the study was the rate of complete wound closure, defined as 100% re-epithelialization without drainage or the need for a dressing. Participants were followed for up to 12 weekly measurements, with follow-up ending earlier in cases of complete wound closure or at the end of the study period. Data were collected through questionnaires and patients’ medical records according to the predefined assessment schedule: T0, before the intervention; T1, the first measurement at 7 days; T2, the second measurement at 14 days; and so on, up to T12, corresponding to the completion of the intervention.

Secondary endpoints included: (1) percentage reduction in wound area, assessed at predefined follow-up intervals; (2) time to complete wound healing (days); (3) incidence of local complications (e.g., surgical site infection, maceration, or device dislodgement); and (4) changes in health-related quality of life, assessed using EuroQol-5D, and pain intensity, assessed using VAS.

The IG patients were treated with the single-use portable NPWT device PICO 7 (Smith & Nephew^®^), selected for its ability to maintain a continuous negative pressure of −80 mmHg, the optimal level to activate tissue repair mechanisms [34]. The device was placed and programmed in continuous mode in the vascular surgery laboratory. Outpatients received clear instructions on how to proceed in case of incidents at home, whereas hospitalized patients continued their treatment in their room. At the seven-day follow-up visit, a new PICO 7 device was placed, as the dressing was often saturated with exudate if the wound had not yet healed. In cases where NPWT was no longer necessary, a transition to conventional care was made. On a weekly basis, participants attended consultations for device replacement and wound care materials. Meanwhile, the CG received conventional treatment following the standardized protocol of Osakidetza [27]. Wounds were cleaned with 0.9% saline solution using an atraumatic technique, and debridement was performed when clinically indicated. Depending on the wound characteristics and exudate level, either antiseptic treatment with povidone-iodine (Betadine) or an appropriate advanced dressing (e.g., hydrocolloids, alginate dressings such as Aquacel, Mepitel, or medical-grade honey) was selected. These approaches were not routinely applied concomitantly. Targeted systemic antibiotic therapy was prescribed exclusively based on culture results and microbiological sensitivity testing and was managed identically in both groups. Compression bandaging was applied for venous ulcers and padding for arterial ulcers [27,43]. In the final measurement, health-related quality of life was reassessed using the EuroQol-5D questionnaire [39].

This study was open-label, as neither participants nor the nurses providing wound care could be blinded to treatment allocation due to the nature of the intervention. Wound assessments and data collection were performed by nurses from the vascular surgery laboratory (CVC, MDBR, ESG). The collected data were subsequently provided to the principal investigator (CVA) without information identifying treatment allocation, allowing the data analysis to be performed without direct knowledge of group assignment.

### 2.8. Data Analysis

For the statistical analysis, the Statistical Package for the Social Sciences (SPSS) version 28 (IBM Corp., Armonk, NY, USA) was used under the license of the University of Castilla-La Mancha (UCLM). Categorical variables were expressed as absolute frequencies and percentages, whereas continuous variables were presented as mean ± standard deviation (SD) or median with interquartile range (IQR) based on normality distribution assessed via the Shapiro–Wilk test.

Due to sample size constraints within individual wound etiology categories, formal subgroup analyses were not conducted to avoid underpowered statistical conclusions. To evaluate wound size progression over time, comparative analyses were performed at discrete, clinically relevant follow-up intervals (baseline, week 4, week 8, and week 12). Between-group comparisons for continuous outcomes at each time point were conducted using Student’s *t*-test or Mann–Whitney U test, whereas categorical endpoint rates were compared using the Chi-square test or Fisher’s exact test. Similarly, a Pearson or Spearman correlation was conducted if they did not follow a normal distribution.

All hypothesis tests were two-tailed. In all statistical tests, values were considered statistically significant when the confidence level was 95% (*p* < 0.05).

### 2.9. Ethical Considerations

This study was approved by the Clinical Research Ethics Committee with drugs of the Hospital Universitario de Toledo with registration number 1102.

The proposed research adhered to the fundamental principles of the Declaration of Helsinki, the Council of Europe’s Convention on Human Rights and Biomedicine, the UNESCO Universal Declaration on Human Rights, Good Clinical Practices (GCP), ISO 14155:2011, and the Oviedo Council concerning Human Rights and Biomedicine.

All participants’ data were treated confidentially, in accordance with Organic Law 3/2018, of 5 December, on Personal Data Protection and the guarantee of digital rights.

## 3. Results

**Figure 1:**
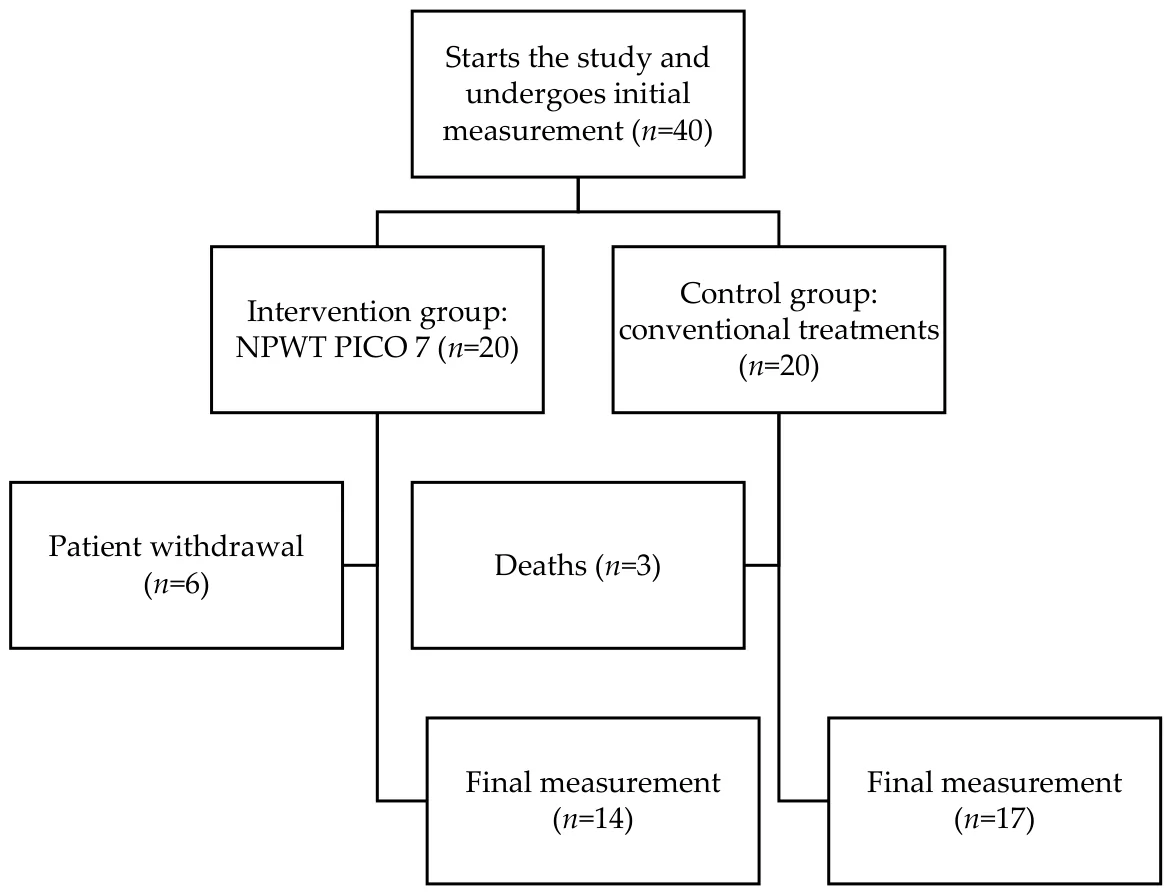
Flowchart of participants.

The flow of participants throughout this study is detailed in Figure 1. The initial sample consisted of 40 participants, assigned through simple randomization into two groups: 20 subjects for the IG and 20 for the CG. No statistically significant differences were observed in baseline characteristics between the two groups (Table 1).

A total of 31 patients (77.5% of the initial sample) completed the follow-up. In the IG, six losses were recorded: four dropouts before the start of the analysis and two subjects who did not complete the treatments due to lack of adherence, resulting in a total of 14 subjects. Meanwhile, in the CG, three deaths were recorded, with 17 subjects completing the study.

Consequently, the final analyzed sample consisted of 31 patients, with a majority of men (78.6%). The etiology and clinical characteristics of the wounds are detailed in Supplementary Table S1, whereas the sociodemographic variables are presented in Table 2.

As shown in Table 2, regarding the categorical variables, no significant differences were found for gender (*p* = 0.612), HBP (*p* = 1.000), or other baseline comorbidities (*p* > 0.05). The only variable that demonstrated a statistically significant difference between nthe groups was dyslipidemia (*p* = 0.024), which was significantly more prevalent in the IG (85.70%) compared to the CG (41.20%).

Baseline sociodemographic and clinical characteristics were generally comparable between the IG (n = 14) and the GC (*n* = 17). No statistically significant differences were observed regarding mean age (*p* = 0.813), BMI (*p* = 0.848), or hours sleep (*p* = 0.397).

### 3.1. Healing Rate, Infections, and Adverse Effects

Regarding the healing rate (Table 3), no significant differences were found between the groups (*p* = 0.637). Regarding adverse effects, there were no significant differences (*p* = 0.442), with only one case of adverse effects in the IG due to a positive “Probe to bone” test on one occasion. With respect to infections, there were no significant differences (*p* = 0.548), with only one case of Staphylococcus Aureus infection in the CG.

### 3.2. Healing Time, Wound Size Reduction, and Pain

**Figure 2:**
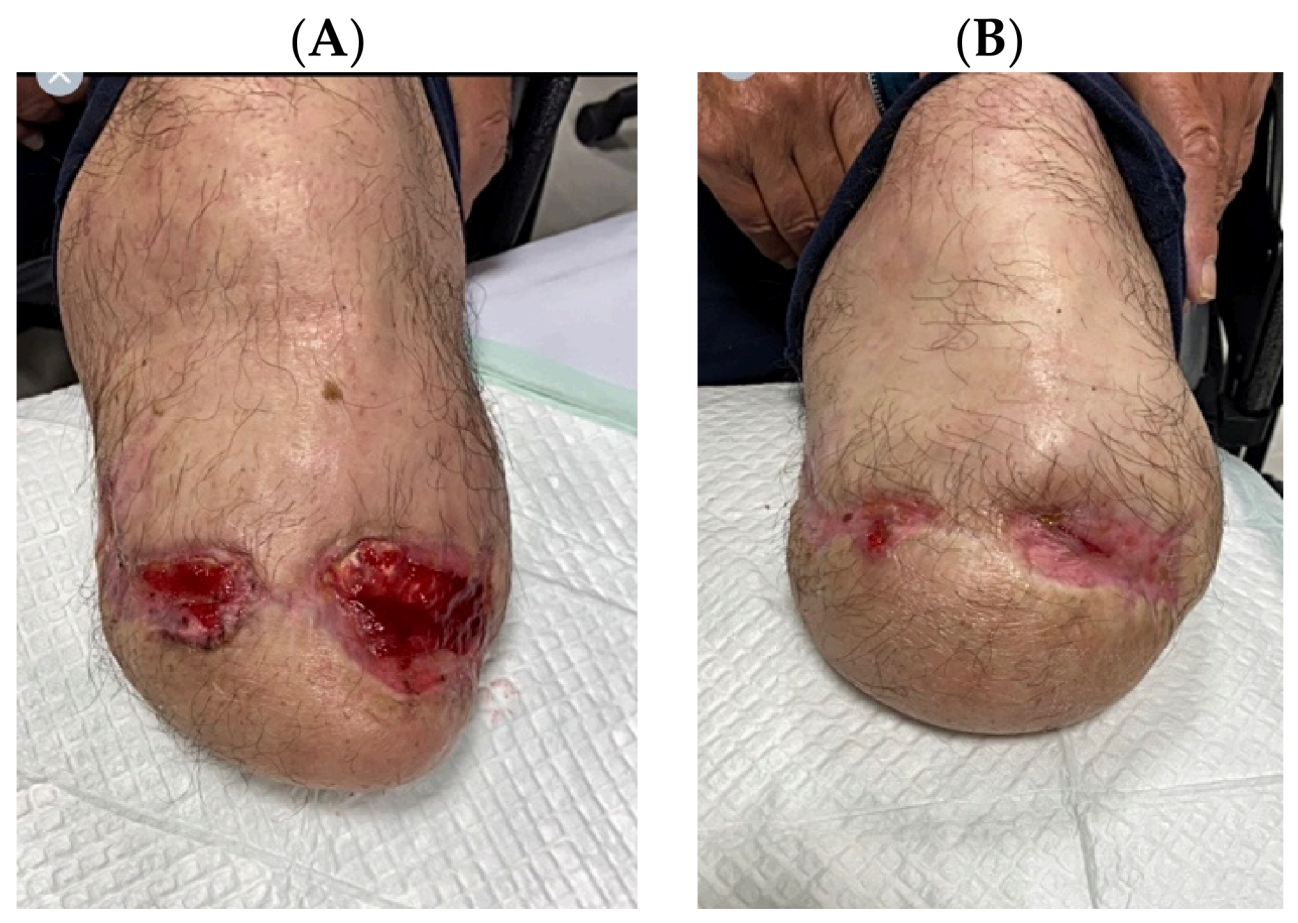
Clinical evolution of surgical wound dehiscence in a transtibial amputation stump treated with single-use NPWT (PICO 7). (**A**) Baseline assessment ($T_{0}$) showing active surgical dehiscence with open wound beds prior to NPWT initiation. (**B**) Final follow-up after PICO 7 therapy, demonstrating complete re-epithelialization, substantial area reduction, and healthy tissue consolidation. Image courtesy of Cristina Del Viso-Cudero.

**Figure 3:**
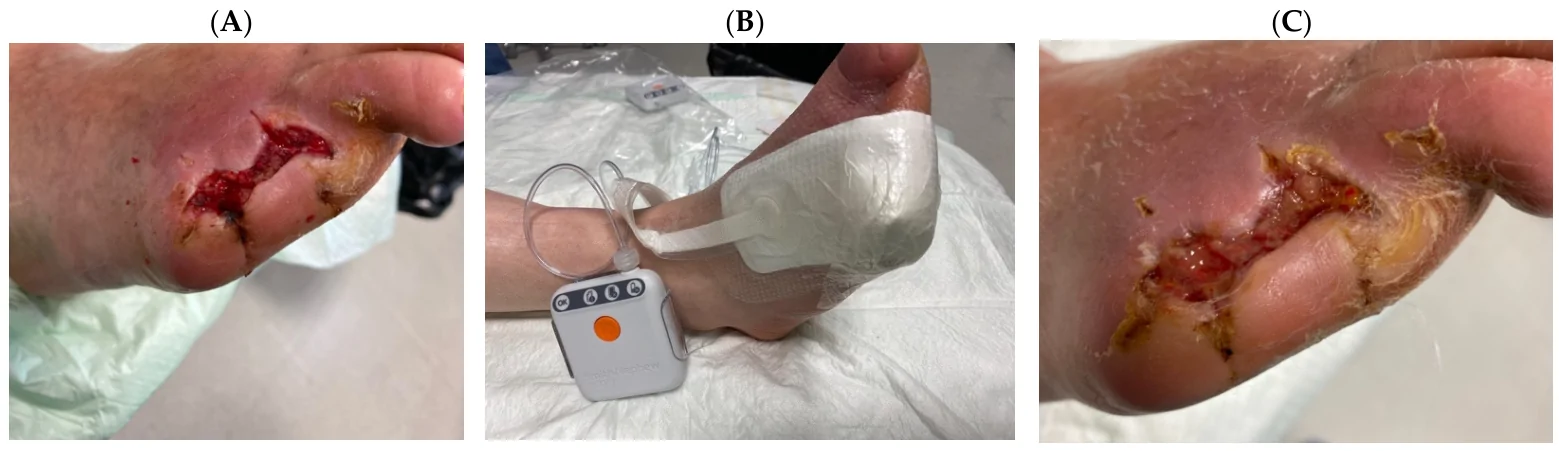
Clinical evolution of a DFU treated with single-use NPWT (PICO 7). (**A**) Baseline assessment (T_0_) showing an open DFU with irregular wound margins and areas of fibrinous tissue. (**B**) Application of the PICO 7 single-use NPWT system to the wound. (**C**) Follow-up assessment after treatment, showing a reduction in wound size, the development of granulation tissue, and progressive wound closure. Image courtesy of Cristina Del Viso-Cudero.

**Figure 4:**
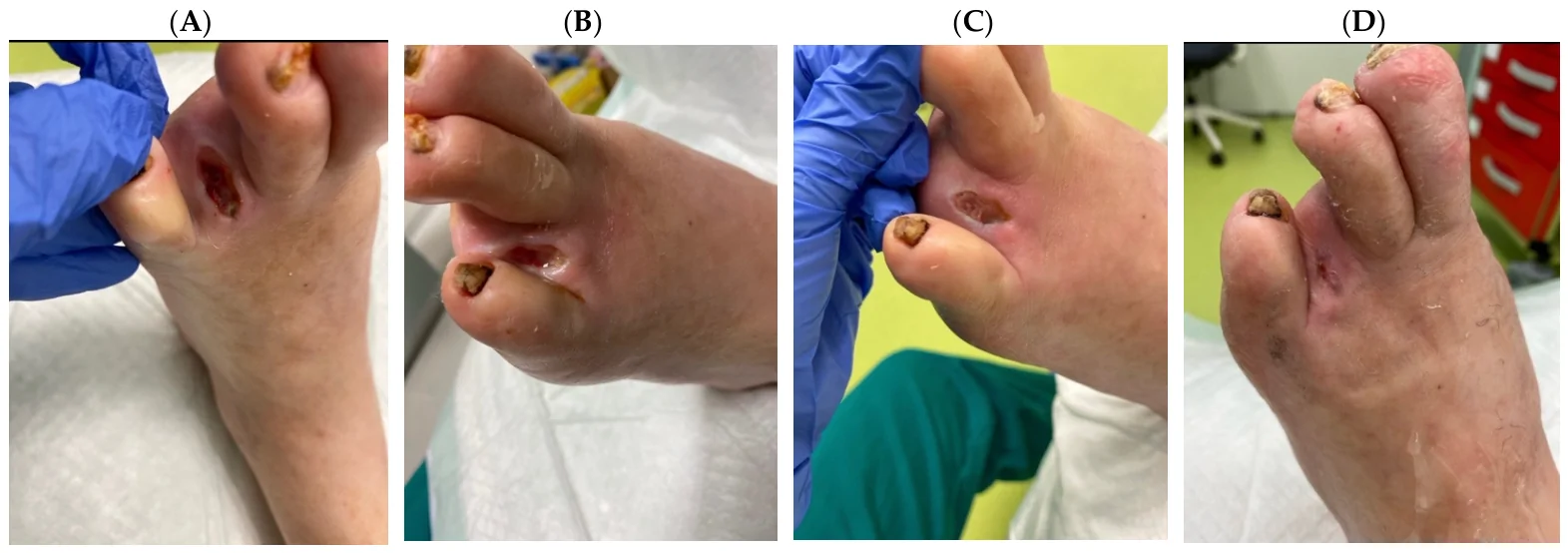
Sequential healing progression of an interdigital DFU managed with single-use NPWT (PICO 7). (**A**) Initial clinical presentation ($T_{0}$) showing an active interdigital diabetic foot ulcer with erythematous perilesional tissue. (**B**) Early follow-up demonstrating exudate control and initiation of granulation tissue formation under PICO 7 therapy. (**C**) Intermediate evaluation exhibiting significant wound contraction, edge stimulation, and the reduction of localized inflammation. (**D**) Final follow-up showing complete re-epithelialization and successful closure of the interdigital diabetic foot ulcer. Image courtesy of Cristina Del Viso-Cudero.

Regarding the size of the wounds, the mean reduction in the IG was 35.70 days (SD = ±6.96), whereas in the CG it was 28.00 (SD = ±19.11), showing no statistically significant difference between the groups (*p* = 0.215). Regarding the reduction in wound size, the IG had a mean percentage reduction of 87.26% (SD = ±26.19) and the CG 75.29 (SD = ±63.65) (*p* = 0.519). Regarding the difference in pain scale scores, the IG had a mean difference of 0.57 (SD = ±1.82) and the CG 1.52 (SD = ±3.41) (*p* = 0.328). None of these continuous outcomes reached statistical significance. These variables are summarized in Table 4. Representative examples of wound evolution during single-use NPWT treatment are depicted in Figure 2A,B, Figure 3A–C and Figure 4A–D.

### 3.3. Differences in Pain, Size, Quality of Life, and Physical Activity

Table 5 shows the differences between pre- and post-intervention outcomes within both groups. Regarding pain scores, no statistically significant intra-group reductions were observed in either the IG (MD=−0.65, 95% CI:−2.29 to 0.99; p=0.417) or the CG (MD=−1.53, 95% CI:−3.18 to 0.12; p=0.067). In contrast, statistically significant reductions in wound size were observed before and after treatment in both groups (p<0.001), with a mean reduction of $-7.18{\;\mathrm{cm}}^{2}$ (95% CI:−10.51 to −3.85) in the IG and $-6.55{\;\mathrm{cm}}^{2}$ (95% CI:−10.25 to −2.85) in the CG. Regarding health-related quality of life (EuroQol-5D), the intra-group change in the IG did not reach statistical significance (MD=−0.93, 95% CI:−1.89 to 0.03; p=0.056), whereas a statistically significant change was observed in the CG (MD=−0.47, 95% CI:−0.82 to −0.12; p=0.011). Finally, regarding physical activity levels (IPAQ), no statistically significant intra-group change was found in the IG (MD=−138.43, 95% CI:−680.15 to 403.29; p=0.593), whereas a statistically significant reduction was observed in the CG (MD=−1294.85, 95% CI:−2256.40 to −333.30; p=0.011).

### 3.4. Effects of HBP, Obesity, Smoking, DM, and Sedentary Lifestyle on Healing Rate and Healing Time

Table 6 shows the influence of baseline cardiovascular and lifestyle factors, including sedentary lifestyle, smoking, DM, HBP, PVD, and obesity, on the healing rate of the participants. No statistically significant association was observed between non-healed (n=9) and healed (n=22) patients for any of the analyzed factors: sedentary lifestyle (*p* = 0.441), HBP (*p* = 1.000), PVD (*p* = 0.077), or obesity (*p* = 1.000).

Table 7 shows the influence of baseline variables—including sedentary lifestyle, smoking, DM, HBP, PVD, and obesity—on wound healing time. Regarding healing time according to the baseline characteristics, no statistically significant differences were observed for sedentary lifestyle (MD=8.28 days, 95% CI:−2.46 to 19.02; p=0.206), smoking status (MD=−11.55 days, 95% CI:−34.25 to 11.15; p=0.313), DM (MD=−11.78 days, 95% CI:−25.21 to 1.65; p=0.128), hypertension (MD=2.14 days, 95% CI:−11.75 to 16.03; p=0.805), or obesity (MD=0.91 days, 95% CI:−12.44 to 14.26; p=0.843).

### 3.5. Correlations

Table 8 presents the bivariate correlation matrix between continuous baseline characteristics and main clinical outcomes (age, BMI, hours of sleep, healing time, final wound size, post-treatment EuroQol-5D, and post-treatment IPAQ). Bivariate correlation analysis revealed no statistically significant correlations among any of the analyzed continuous variables (p>0.05 for all comparisons). Weak-to-moderate non-significant correlations were observed, such as a negative trend between hours of sleep and final wound size (r=−0.470,  p>0.05), BMI and post-treatment EuroQol-5D (r=−0.299,  p>0.05), and a weak positive trend between healing time and post-treatment IPAQ scores (r=0.284,  p>0.05).

## 4. Discussion

The findings of this trial demonstrate a favorable clinical trend in the IG regarding percentage wound area reduction and zero incidence of local infections; however, these differences did not reach statistical significance compared to conventional therapy. Rather than proving superiority or confirming accelerated healing speed or reduced infection risk, our results indicate that single-use portable NPWT (PICO 7) represents a feasible, safe, and comparable therapeutic alternative in real-world outpatient practice. A major consideration in evaluating these findings is the marked pathophysiological heterogeneity of the study sample, which included DFU, venous and arterial ulcers, and post-amputation surgical dehiscence. Because different chronic wound etiologies exhibit distinct microvascular environments, tissue perfusion dynamics, and intrinsic healing capacities, NPWT may exert varying therapeutic effects across different lesion types. This biological diversity, combined with our small analytical sample size (n = 31), limits the direct generalizability of our findings to single-etiology populations and explains why favorable clinical trends did not achieve statistical significance. These results align with previous observations by Seidel et al. [44], who similarly found no statistically significant differences in closure rates among diabetic foot ulcers, largely attributable to sample size constraints. In contrast, larger trials [45,46,47] have demonstrated statistically significant advantages for NPWT. Given our reduced final sample size (n = 31), the present study lacks the statistical power to detect minor between-group differences, and our observations must be interpreted as favorable clinical trends rather than definitive statistical proof.

Regarding healing time, the IG showed a more homogeneous evolution than the CG. Although some authors reported significant reductions in healing time in DFUs [46,48], in our work and coinciding with the study by Braakenburg et al. [49], no significant differences were observed between NPWT and conventional dressings in chronic wounds. By including wounds of diverse etiology (from VFU to major amputation dehiscence), the impact of NPWT on closure speed might appear less striking than in samples that only analyze one type of lesion. Although the IG showed less dispersion in healing times, this finding should be interpreted descriptively, as no statistically significant difference in healing time was observed between the groups (*p* = 0.215). Further studies with larger samples are needed to determine whether this apparent difference in variability represents a clinically meaningful effect [50,51,52,53].

Regarding wound size reduction, although the IG demonstrated a higher mean percentage reduction, this difference was not statistically significant (*p* = 0.519) and must be interpreted cautiously as a favorable descriptive trend rather than evidence of a treatment effect [50,54,55]. Similarly, although reduced dressing change frequency and continuous negative pressure are clinically hypothesized to minimize dressing disruption and local irritation [56], pain score reductions (*p* = 0.328) and quality of life measures (*p* = 0.056) did not differ significantly between the groups [54,57,58,59]. Furthermore, because formal health economic parameters and treatment-related costs were not evaluated in this study, no conclusions regarding economic efficiency or cost-effectiveness can be drawn. Consequently, single-use NPWT should be considered a safe, feasible, and clinically comparable alternative to conventional care in outpatient settings, rather than a superior or cost-saving intervention.

An analysis of the impact of baseline risk factors showed that comorbidities such as DM or obesity did not prevent wound progress under the study protocol. Although previous literature [6,14] hypothesizes that NPWT may support tissue granulation and the microvascular response through mechanical strain and exudate management, our trial did not measure histological markers or microvascular perfusion; thus, the potential angiogenic mechanism remains theoretical in this context. Similarly, although reduced dressing change frequency and stable sub-atmospheric pressure may minimize pain peaks during wound handling, any potential indirect benefits on overall patient comfort or sleep patterns remain speculative and warrant direct objective evaluation in future research [60,61]. On the other hand, PVD was the factor closest to significance, achieving 100% success in wounds of venous etiology, likely because NPWT accelerates granulation in more superficial beds [62].

Crucially, conservative wound care technologies such as single-use NPWT should not be conceptualized as isolated monotherapies, but rather as integral components of a comprehensive, multidisciplinary limb-salvage strategy. In modern diabetic foot and complex lower extremity management, topical advanced therapies must be synchronized with surgical interventions. This includes thorough surgical debridement, targeted revascularization procedures when macrovascular ischemia is present, and reconstructive surgical strategies. Specifically, surgical off-loading techniques (such as distal metatarsal osteotomies (DMOs), tenotomies, or exostectomies) play a pivotal complementary role by structurally correcting underlying mechanical deformities and relieving pathological plantar pressure peaks. When integrated within a multidisciplinary framework combining surgical correction, appropriate biomechanical off-loading, and advanced wound bed preparation, single-use NPWT may contribute to wound bed management and progression toward definitive closure; however, these potential mechanisms were not directly assessed in the present study [63,64,65,66,67].

On the other hand, a favorable trend was observed regarding healing time, being shorter in those who engaged in more physical activity. This trend is consistent with the study by Qiu et al. [68] who, in a prospective analysis, observed that higher levels of physical activity are associated with a greater probability of healing in patients with VFU. Similarly, there was a predisposition toward longer closure times in smoking patients. This coincides with a systematic review and meta-analysis, in which it was observed that smoking increases the risk of complications and prolongs closure times due to the vasoconstriction caused by nicotine and the reduction of oxygen transport to tissues [69].

It is worth noting that the absence of statistically significant differences in some healing parameters may also reflect the high level of wound care provided to participants in the CG. In our setting, nursing professionals have advanced training in the management of complex wounds, which may have reduced the potential between-group differences [70].

On the other hand, the correlation analysis of the total sample allowed identifying some possible clinical determinants of the recovery process, suggesting that closing the lesion facilitates better rest by reducing exudate and pain, results that coincide with the study by Upton et al. [71], who found that patients with chronic ulcers in the lower limbs presented with sleep problems.

Therefore, the PICO 7 system has proven to be a safe and reliable option, as it does not increase complications or is associated with an increase in adverse events or infections. Its competitive advantage lies in its outpatient nature, which allows maintaining an active lifestyle. Clinically, the reduction in wound surface area leads to healthcare savings of up to 33% by optimizing nursing time and reducing the use of conventional dressings [22]. Following the line of Ibáñez et al. [72], by integrating into their daily environment, not only is self-care promoted, but the psychological burden of chronicity is alleviated, resulting in a substantial improvement in their quality of life.

A critical consideration in interpreting our findings is the marked pathophysiological heterogeneity of this study. By evaluating DFUs, venous leg ulcers, arterial ulcers, and post-amputation surgical dehiscence concurrently, this trial reflects a real-world pragmatic clinical setting. However, these conditions represent distinct biological entities with varying microvascular status, underlying tissue perfusion, reconstructive needs, and intrinsic healing potential. This biological diversity inevitably introduces confounding variability, which constrained our ability to perform stratified subgroup analyses without losing statistical power, thereby obscuring the potential etiology-specific therapeutic effects of NPWT. Although our pragmatic design supports broad applicability across general outpatient vascular nursing consultations, future multicenter trials with larger cohorts should stratify by specific ulcer etiologies to establish tailored treatment protocols [73].

Regarding the remaining limitations of this study, several factors must be considered. First, although an adequate sample size was calculated at baseline, participant dropouts and mortality reduced the final analytical cohort. This loss to follow-up decreased statistical power, which may explain why certain favorable clinical trends did not reach statistical significance. Second, the open-label nature of the trial was unavoidable, as the physical characteristics of the PICO 7 system prevent blinding for both clinicians and patients, introducing an inherent source of performance bias.

Third, patient-reported outcome measures, including health-related quality of life (EuroQol-5D) and physical activity levels (IPAQ), were assessed via self-reported questionnaires within this unblinded framework. Consequently, these subjective endpoints are inherently vulnerable to expectation, social desirability, and recall biases, as patients receiving the active technological device may have perceived an enhanced sense of care. Finally, although the follow-up duration was sufficient to observe primary closure in most lesions, an extended observation period would be necessary to accurately evaluate long-term outcomes, such as wound recurrence rates. Also, the study was also limited by participant attrition, which reduced the final sample size and consequently the statistical power of the analyses. The Per-Protocol approach may have introduced attrition bias, particularly given the higher dropout rate in the intervention group. Therefore, the findings should be interpreted cautiously and considered preliminary.

However, this study presents diagnostic and methodological strengths of great relevance. It is the first clinical trial conducted in Spain that evaluates the efficacy of the PICO 7 system in chronic wounds of the lower limbs, providing evidence in our healthcare setting. The rigor of the protocol is supported by its prior registration in ClinicalTrials.gov, ensuring data transparency. Additionally, the baseline homogeneity achieved between the IG and CG reinforces the internal validity of the comparison.

## 5. Conclusions

The single-use portable NPWT system (PICO 7) represents a safe clinical option with potential clinical benefits for patients with lower limb chronic wounds and post-amputation surgical dehiscence. Given the sample size constraints, this study does not demonstrate statistical superiority over conventional wound care. Observed parameters such as wound area reduction trends and low local complication rates within the IG must be interpreted with appropriate caution strictly as preliminary clinical trends rather than definitive statistical evidence.

Regarding patient safety and pain management, the PICO 7 system demonstrated good clinical tolerability, although no statistically significant between-group difference was observed in pain reduction.

Regarding comorbidities, baseline conditions such as DM, obesity, and smoking did not prevent progress under the study protocol in a real-world clinical practice setting, highlighting the system’s pragmatic feasibility in outpatient nursing care.

For future research, larger multicenter RCTs with adequate statistical power and stratification according to specific wound etiologies (e.g., DFUs, venous leg ulcers, and surgical dehiscence) are needed to further validate the clinical effectiveness and formal economic profile of single-use NPWT systems. Additionally, extended follow-up periods are recommended to evaluate long-term recurrence rates and confirm the durability of wound closure.

## Figures and Tables

**Table 1:** Overview of the study design, intervention protocols, and outcome measures.

Component	Description
Study design and setting	Randomized, open-label clinical trial conducted at Hospital Universitario de Toledo (Jan 2024–Jan 2026).
Study Population	Adults (≥18 years) with lower-limb chronic wounds (DFU, VFU, or post-amputation dehiscence < 20 cm^2^).
IG	Single-use portable NPWT (PICO 7) in continuous mode + dynamic TIME wound bed preparation.
CG	Standardized conventional care protocol (Osakidetza care path: Betadine, advanced dressings, compression/padding) + TIME strategy.
Main outcomes	Healing rate, healing time (days), wound area reduction (% and cm^2^), local infections, pain scores, EuroQol-5D, and IPAQ.
Statistical Analysis	Chi-square/Fisher’s exact test for categorical variables; Student’s *t*-test/Mann–Whitney U for continuous variables (*p* < 0.05).

**Table 2:** Sociodemographic characteristics of the participants.

Sociodemographic Characteristics	Intervention Group (*n* = 14) *n* (%)	Control Group (*n* = 17) *n* (%)	*p*-Value
*Gender*			0.612 *
Male	11 (78.60)	12 (70.60)	
Female	3 (21.40)	5 (29.40)	
*Sedentary lifestyle*			0.332 *
Yes	9 (64.30)	8 (47.10)	
No	5 (35.70)	9 (52.90)	
*Rest*			
Very good	5 (35.70)	1 (5.90)	0.621 *
Good	8 (57.10)	13 (76.50)	
Regular	1 (7.10)	2 (11.80)	
Poor	0 (0.00)	1 (5.90)	
*Diet*			0.637 *
Balanced and healthy	11 (78.60)	15 (88.20)	
Fair	3 (21.4)	2 (11.80)	
*Smoker*			1.000 *
Yes	13 (92.90)	16 (94.10)	
No	1 (7.10)	1 (5.90)	
*DM*			1.000 *
Yes	11 (78.60)	14 (82.40)	
No	3 (21.40)	3 (17.60)	
HBP			1.000 *
Yes	11 (78.60)	14 (82.40)	
No	3 (21.40)	3 (17.60)	
*DL*			0.024 *
Yes	12 (85.70)	7 (41.20)	
No	2 (14.30)	10 (58.80)	
*PVD*			1.000 *
Yes	13 (92.90)	16 (94.10)	
No	1 (7.10)	1 (5.90)	
*CHF*			0.669 *
Yes	4 (28.60)	3 (17.60)	
No	10 (71.40)	14 (82.40)	
*Rheumatoid arthritis*			1.000 *
Yes	1 (7.10)	1 (5.90)	
No	13 (92.90)	16 (94.10)	
*Osteoarthritis*			0.113 *
Yes	0 (0.00)	4 (23.50)	
No	14 (100.00)	13 (76.50)	
*Varicose veins*			0.608 *
Yes	1 (7.10)	3 (17.60)	
No	13 (92.90)	14 (82.40)	
*Anemia*			1.000 *
Yes	2 (14.30)	2 (11.80)	
No	12 (85.70)	15 (88.20)	
*Neuropathy*			0.720 *
Yes	5 (35.70)	5 (29.40)	
No	9 (64.30)	12 (70.60)	
	**Intervention Group (n = 14)** **Mean ± SD**	**Control Group (n = 17)** **Mean ± SD**	***p*-Value**
*Mean age*	70.29 (±6.00)	70.94 (±8.69)	0.813
*BMI*	27.48 (±4.00)	27.18 (±4.45)	0.848
*Hours of sleep*	8.43 (±0.85)	8.00 (±1.69)	0.397

Abbreviations: n: sample size; *p*: statistical significance level; DM: diabetes mellitus; HBP: hypertension; DL: dyslipidemia; PVD: peripheral vascular disease; CHF: heart failure; SD: standard deviation; BMI: body mass index. * *p*-values for categorical variables were calculated using Fisher’s exact test. *p*-values and 95% Confidence Intervals for continuous variables (Mean age, BMI, Hours of sleep) were calculated using Independent Sample Student’s *t*-tests.

**Table 3:** Healing rate, infections, and adverse effects for participants.

	Control Group (*n* = 17) *n* (%)	Intervention Group (*n* = 14) *n* (%)	*p*-Value
*Healing*			0.637
No	5 (29.4)	4 (28.57)	
Yes	12 (70.6)	10 (71.42)	
*Adverse effects*			0.452
No	17 (100.0)	13 (92.86)	
Yes	0	1 (7.14)	
Infection at any time			0.548
No	16 (94.12)	14 (100.00)	
Yes	1 (6.25)	0 (0.00)	

**Table 4:** Variables of participants’ wounds.

Wound Variables	Intervention Group (*n* = 14) m (SD)	Control Group (*n* = 17) m (SD)	Mean Difference (95%CI)	*p*-Value
*Healing Time*	35.70 (±6.96)	28.00 (±19.11)	7.70 (−3.11 to 18.51)	0.215
*Size Reduction*	87.26% (±26.19)	75.29 (±63.65)	11.97 (−25.59 to 49.53)	0.519
*Pain Difference*	0.57 (±1.82)	1.52 (±3.41)	−0.95 (−2.91 to 1.01)	0.328

**Abbreviations:** m: mean; SD: standard deviation; CI: confidence interval.

**Table 5:** Differences between pre- and post-intervention between the participants of both groups.

		Intervention Group				Control Group		
	Pre-Test	Post-Test	MD (95% CI)	*p*-Value	Pre-Test	Post-Test	MD (95% CI)	*p*-Value
	m (SD)	m (SD)			m (SD)	m (SD)		
*Pain*	4.36 (±3.50)	3.71 (±3.52)	−0.65 (−2.29 to 0.99)	0.417	4.71 (±1.96)	3.18 (±3.05)	−1.53 (−3.18 to 0.12)	0.067
*Size*	7.75 (±5.64)	0.57 (±1.59)	−7.18 (−10.51 to −3.85)	<0.001	7.44 (±7.09)	0.89 (±2.46)	−6.55 (−10.25 to −2.85)	<0.001
*EuroQol-5D*	9.28 (±1.27)	8.35 (±1.90)	−0.93 (−1.89 to 0.03)	0.056	8.29 (±2.47)	7.82 (±2.50)	−0.47 (−0.82 to −0.12)	0.011
*IPAQ*	572.14 (±1060.23)	433.71 (±929.41)	−138.43 (−680 to 403. 29)	0.593	3554.02 (±10,292.84)	2259.17 (±8389.90)	−1294.85 (−2256.40 to 333.30)	0.011

Note: *p*-values and 95% Confidence Intervals for within-group changes (Post-test minus Pre-test) were calculated using Paired Sample Student’s *t*-tests. **Abbreviations:** EuroQol-5D: health-related quality of life measurement questionnaire; IPAQ: levels of physical activity measured by calculating metabolic index (Mets) units; m: mean; SD: standard deviation; CI: confidence interval.

**Table 6:** Effect of sedentary lifestyle, smoking, DM, HBP, PVD, and obesity on healing rate.

Sociodemographic Variables	Non-Healed *n* (%)	Healed *n* (%)	*p*-Value
*Sedentary lifestyle*			0.441 *
Yes	6 (66.7)	11 (50.0)	
No	3 (33.3)	11 (50.0)	
*Smoker*			1.000 *
Yes	0 (0.00)	2 (9.1)	
No	9 (100.0)	20 (90.9)	
*DM*			00.643 *
Yes	8 (88.9)	17 (77.3)	
No	1 (11.1)	5 (22.7)	
*HBP*			1.000 *
Yes	7 (77.8)	18 (81.8)	
No	2 (22.2)	4 (18.2)	
*PVD*			0.077 *
Yes	7 (77.8)	22 (100.0)	
No	2 (22.2)	0 (0.0)	
*Obesity*			1.000 *
Yes	7 (77.8)	17 (77.3)	
No	2 (22.2)	5 (22.7)	

* *p*-values were calculated using Fisher’s exact test. **Abbreviations:** DM: diabetes mellitus; HBP: hypertension; PVD: peripheral venous disease.

**Table 7:** Effect of sedentary lifestyle, smoking, DM, HBP, PVD, and obesity on healing time.

Sociodemographic Variables	Healing Time m (SD)	Mean Difference (95% CI)	*p*-Value
*Sedentary Lifestyle*			
Yes	35.64 (13.07)	8.28 (−2.46 to 19.02)	0.206
No	27.36 (16.40)		
*Smoker*			
Yes	21.0 (9.89)	−11.55 (−34.25 to 11.15)	0.313
No *DM*	32.55 (15.281)		
Yes	28.82 (15.23)	−11.78 (−25.21 to 1.65)	0.128
No	40.60 (11.50)		
*HBP*			
Yes	31.89 (16.32)	2.14 (−11.75 to 16.03)	0.805
No	29.75 (8.80)		
Obesity			
Yes	31.71 (17.16)	0.91 (−12.44 to 14.26)	0.843
No	30.80 (3.83)		

Note: Healing time is reported in days. Mean differences (Yes minus No) and 95% Confidence Intervals were calculated using Independent Sample Student’s *t*-tests. **Abbreviations:** DM: diabetes mellitus; HBP: hypertension; m: mean; SD: standard deviation.

**Table 8:** Correlations of study variables (age, BMI, sleep, healing, quality of life, and physical activity).

	Age	BMI	Hours of Sleep	Healing Time	Final Size	EuroQol5D-Post	IPAQ-Post
*Age*	-	−0.004	0.078	0.163	0.189	−0.106	−0.047
*BMI*		-	0.305	−0.176	−0.079	−0.299	0.027
*Hours of sleep*			-	0.216	−0.470	−0.069	−0.170
*Healing time*				-	-	−0.180	0.284
*Final size*					-	0.212	−0.071
*EuroQol-5D-post*						-	−0.197
*IPAQ-post*							*-*

**Abbreviations:** BMI: body mass index; EuroQol-5D-post: health-related quality of life measurement questionnaire after treatment; IPAQ-post: levels of physical activity measured through calculation of the metabolic index units (Mets) after treatment.

## Data Availability

All data are available from the corresponding author upon reasonable request. Throughout the study process and in the future, the anonymity of all participants is guaranteed.

## Supplementary Materials

The following supporting information can be downloaded at: https://www.mdpi.com/article/10.3390/jcm15166373/s1, Table S1: Baseline wound characteristics.

## Abbreviations

BMI	Body mass index
CHF	Congestive heart failure
CI	Confidence interval
CVD	Chronic venous disease
CVI	Chronic venous insufficiency
DFU	Diabetic foot ulcer
DL	Dyslipidemia
DM	Diabetes Mellitus
DMO	Distal metatarsal osteotomies
DVT	Deep vein thrombosis
FDA	Food and drug administration
HBP	Hypertension
IPAQ	International Physical Activity Questionnaire
LL	Lower limb
MD	Mean difference
NICE	National Institute for Health and Care Excellence
NPWT	Negative pressure wound therapy
PAD	Peripheral arterial disease
PRP	Platelet-rich plasma
PVD	Peripheral vascular disease
RCT	Randomized controlled trial
VFU	Vascular foot ulcer
