# Supplementary material for: Effectiveness of Single-Use Negative Pressure Wound Therapy (Pico 7) in Chronic Lower Limb Wounds: A Randomized Clinical Trial
Source: J Clin Med. 2026 Aug 18;15(16):6373. doi: 10.3390/jcm15166373 (PMC13513595; doi:10.3390/jcm15166373)
Supplement: Supplementary file 1 [file jcm-15-06373-s001.zip › jcm-4468993-supplementary.pdf]

Supplementary Table S1: Baseline wound characteristics.

Wound	Etiology	Localization	Initial wound area (cm^2)
1	Surgical wound (infracondylar amputation)	Front of right knee	20.00
2	DFU (amputation)	4th toe of left foot	2.00
3	Surgical wound (infracondylar amputation)	Front of left knee	17.00
4	Surgical wound (amputation)	Front of left knee	2.00
5	Surgical wound (trans-metatarsal amputation)	Left foot	5.00
6	Surgical wound (supracondylar amputation)	Left leg	4.00
7	DFU	Malleolus inner left foot	4.00
8	DFU	Malleolus inner right foot	2.00
9	DFU (amputation)	3rd, 4th and 5th toes of right foot	20.00
10	Surgical wound (trans-metatarsal amputation)	1st right foot	15.00
11	Venous ulcer	Inner left leg	20.00
12	Venous ulcer	Inner lateral right leg	8.00
13	Surgical wound (dehiscence)	Right pelvis	10.00
14	DFU (metatarsal amputation)	Left foot	9.00
15	Surgical wound (amputation infracondylar)	Right foot	15.00
16	Surgical wound (infracondylar dehiscence amputation)	Anterior right knee	5.00
17	Venous ulcer	Heel of left foot	1.00
18	Surgical wound (dehiscence)	Right hip	8.00
19	DFU (amputation)	2nd toe, right foot	3.00
20	Surgical wound (amputation trans-metatarsal)	Right foot	1.50
21	Venous ulcer	Shin, left leg	10.00
22	Venous ulcer	Inner right leg	12.00
23	Venous ulcer	Pretibial area, right leg	8.00
24	DFU (amputation)	3rd toe, right foot	1.50
25	Surgical wound (supracondylar amputation)	Right leg	10.00

26	DFU	Heel, left foot	1.50
27	Surgical wound (dehiscence)	Lateral left leg	20.00
28	Surgical wound	Inguinal area, right leg	10.00
29	DFU	Lateral malleolus, right foot	2.00
30	Surgical wound (infracondylar amputation)	Anterior left knee	6.00
31	DFU	Lateral ankle area, right foot	0.50
32	DFU	5th toe, left foot	2.00
33	Surgical wound (supracondylar amputation)	Left leg	2.00
34	Arterial ulcer	Pretibial area, right leg	3.00
